# Development and validation of a carotid plaque risk prediction model for coal miners

**DOI:** 10.3389/fcvm.2025.1490961

**Published:** 2025-05-09

**Authors:** Yi-Chun Li, Tie-Ru Zhang, Fan Zhang, Chao-Qun Cui, Yu-Tong Yang, Jian-Guang Hao, Jian-Ru Wang, Jiao Wu, Hai-Wang Gao, Ying-Bo Liu, Ming-Zhong Luo, Li-Jian Lei

**Affiliations:** ^1^ Department of Epidemiology, School of Public Health, Shanxi Medical University, Taiyuan, Shanxi, China; ^2^ MOE Key Laboratory of Coal Environmental Pathogenicity and Prevention, Shanxi Medical University, Taiyuan, Shanxi, China; ^3^ Research Centre of Environmental Pollution and Major Chronic Diseases Epidemiology, Shanxi Medical University, Taiyuan, Shanxi, China; ^4^ Department of Occupational Diseases and Poisoning, The Second People’s Hospital of Shanxi Province, Taiyuan, China; ^5^ Department of Medical and Education, The Second People’s Hospital of Shanxi Province, Taiyuan, China; ^6^ Peking University Medical Lu'an Hospital Health Management Center, Changzhi, Shanxi, China; ^7^ Office of the President, The Second People’s Hospital of Shanxi Province, Taiyuan, China

**Keywords:** XGBoost, nomogram, machine learning, coal miners, carotid plaque

## Abstract

**Objective:**

Carotid plaque represents an independent risk factor for cardiovascular disease and a significant threat to human health. The aim of the study is to develop an accurate and interpretable predictive model for early detection the occurrence of carotid plaque.

**Methods:**

A cross-sectional study was conducted by selecting coal miners who participated in medical examinations from October 2021 to January 2022 at a hospital in North China. The features were initially screened using extreme gradient boosting (XGBoost), random forest, and LASSO regression, and the model was subsequently constructed using logistic regression. The three models were then compared, and the optimum model was identified. Finally, a nomogram was plotted to increase its interpretability.

**Results:**

The XGBoost algorithm demonstrated superior performance in feature screening, identifying the top five features as follows: age, systolic blood pressure, low-density lipoprotein cholesterol, white blood cell count, and body mass index (BMI). The area under the curve (AUC), sensitivity, and specificity of the model constructed based on the XGBoost algorithm were 0.846, 0.867, and 0.702, respectively.

**Conclusions:**

It is possible to predict the presence of carotid plaque using machine learning. The model has high application value and can better predict the risk of carotid artery plaque in coal miners. Furthermore, it provides a theoretical basis for the health management of coal miners.

## 1 Introduction

Carotid plaque is an independent risk factor for stroke (1), coronary heart disease (2), and atherosclerotic cardiovascular disease (3). These diseases are the major cause of disability and death globally (4, 5), and they pose a serious economic burden in both developed and developing countries (6). A study has shown that the prevalence of carotid plaque in the middle-aged and elderly population in China is 60.3% (7). As the population of China continues to age and urbanize, the prevalence of cardiovascular and cerebrovascular diseases is expected to increase (8). In some research, occupational stress is identified as a risk factor for cardiovascular disease, with the potential for atherosclerosis to develop as a result of long-term occupational stress (9). China is the world's largest coal producer, with 95% of its coal sourced from underground mining (10), which employs over six million workers (11). In comparison to the general population, coal miners are exposed to a number of harmful factors, including dust (12) and shift work (13). Additionally, they tend to engage in a range of adverse lifestyle habits, such as smoking (14)and alcohol consumption, which can increase the likelihood of developing carotid plaques. Therefore, it is crucial to implement early screening and intervention strategies for coal miners to delay the occurrence and progression of carotid plaque. The majority of studies to date have focused on disease risk in the general population (15, 16), with fewer studies investigating the prediction of disease risk in coal miners.

Predictive models can estimate the probability or risk of an outcome using the characteristics of an individual (17), which is called a diagnostic model. Diagnostic models are of crucial significance in healthcare. They reduce testing costs, enhance the accuracy, efficiency and objectivity of decision-making. They facilitate clinicians in diagnosing and treating patients more effectively, thereby improving the quality of healthcare and the patient care experience.

This study used extreme gradient boosting (XGBoost), random forests, and LASSO regression to filter features for predicting carotid plaque risk in coal miners. Then, logistic regression was applied to create a simple practical risk prediction model to identify at-risk individuals.

## 2 Material and methods

### 2.1 Data sources and subjects

Data were retrospectively collected from coal miners who attended physical examinations at a hospital in North China between October 2021 and January 2022. After excluding incomplete data recorders, attendees younger than 18 years or older than 60 years, and participants with cardiovascular disease, 2,956 participants were included in the study for the prediction model.

### 2.2 Ethical approval

All procedures were approved by the Research Ethics Committee of the Second People's Hospital of Shanxi Province and were conducted strictly in accordance with internationally recognized ethical standards for human research. All participants in this survey were aware of the research contents and precautions and participated voluntarily.

### 2.2 Potential predictors and case definition

A review of the pertinent literature on carotid plaque and an analysis of the accessibility of predictors led to the identification of 27 potential factors: (1) Demographic characteristics: gender, age; (2) Physical examination indicators: height (HT), weight (WT), body mass index (BMI), systolic blood pressure (SBP), diastolic blood pressure (DBP); (3) Laboratory tests: total cholesterol (TC),triglycerides (TG), high-density lipoprotein cholesterol (HDL-C), low-density lipoprotein cholesterol (LDL-C), fasting blood glucose (FBG), alanine aminotransferase (ALT), aspartate aminotransferase (AST), direct bilirubin (DBIL), total bilirubin (TBIL), alkaline phosphatase (ALP), uric acid (UA), platelet count (PLT), white blood cell count (WBC), creatinine (CRE); (4) Lifestyle habits: smoking, alcohol consumption; (5) Occupational factors: years of working experience, exposure to dust (rock dust and coal dust), exposure to hazardous gases (carbon monoxide and hydrogen sulfide); (6) Other indicators: fatty liver disease (FLD).

Cases were defined as whether participants were diagnosed with carotid plaque by carotid ultrasound. The diameters and IMT of the distal common carotid artery, the carotid bulb, and the proximal internal carotid artery were measured within 1–1.5 cm below the level of the bifurcation of the participant's internal and external carotid arteries by an experienced physician to observe the presence of atherosclerotic plaque.

### 2.3 Data processing and predictive modelling

To make full use of the data and evaluate the model's performance, we first randomly divided the dataset into a training set (70%) and a test set (30%), which were used for model training. To ensure the robustness and generalizability of our model, all the data in the training set were utilized in ten-fold cross-validation for model training.

The statistical analyses in this study were conducted using IBM SPSS 26.0. The data, which exhibited a normal distribution, were expressed as “`x ± s”, and t-tests were employed for comparisons between groups. The data, which did not exhibit a normal distribution, were expressed as “[M (P_25_, P_75_)]”, and rank-sum tests were employed for comparisons between groups. The data for categorical variables were expressed as percentages, and the Pearson *χ*^2^ test was employed for comparisons between groups. The level of the test was set at *α* = 0.05 in this paper. R4.2.3 was employed for the purpose of feature screening, model construction, the generation of nomograms, and the assessment of the effects.

XGBoost, random forest, and LASSO regression were used to select the features from the training set as input variables, and the incidence of carotid artery plaque as the output variable to construct a logistic regression model. Considering the potential multicollinearity among different variables, which may lead to model instability, the study evaluated the features selected by three machine learning algorithms based on correlation statistical charts as part of model selection. The performance of the three models was compared by the area under the ROC curve (AUC), net reclassification index (NRI), and integrated discriminant improvement index (IDI), and ultimately selected the model with the best performance. Subsequently, the optimal model was used to construct a nomogram, which was evaluated using decision curves (DCA) and clinical impact curves (ICI).

## 3 Results

### 3.1 Basic characteristics of the study object

A total of 2,956 individuals were included in this study, with a prevalence of carotid plaque of 10.52%. Patients who developed carotid plaque were older and had a higher prevalence in men compared to the no carotid plaque group. They also had higher BMI, systolic blood pressure, diastolic blood pressure, total cholesterol, triglycerides, LDL cholesterol, fasting blood glucose, alkaline phosphatase, white blood cell count, creatinine, years of working experience, a higher prevalence of fatty liver disease, and higher rates of alcohol and smoking. The statistical analysis revealed that there were significant differences in the predictors between healthy individuals and patients with carotid plaque (Table 1). Following the random allocation of the data, a total of 2,069 individuals were included in the training set, with a prevalence of carotid plaque of 10.54% (Supplementary Table S1), and a total of 887 individuals were included in the test set, with a prevalence of carotid plaque of 10.48% (Supplementary Table S2).

**Table 1:** Basic characteristics of the study population.

Characteristic	Carotid plaque		*P* value
	Yes (*n* = 311)	No (*n* = 2,645)	
Sex, *n* (%)			<0.001
Female	34 (10.9%)	744 (28.1%)	
Male	277 (89.1%)	1,901 (71.9%)	
Age (years), (mean ± SD)	47.5 ± 6.53	38.6 ± 7.73	<0.001
HT (cm), median (IQR)	169.50 (164.90–174.00)	170.10 (164.40–175.10)	0.774
WT (kg), median (IQR)	76.00 (68.20–83.00)	17.70 (64.80–82.40)	0.004
BMI (kg/m^2^), median (IQR)	26.34 (24.41–28.36)	25.52 (23.21–27.82)	<0.001
SBP (mm/Hg), mean (SD)	143.00 (19.10)	132.00 (16.60)	<0.001
DBP (mm/Hg), mean (SD)	86.00 (13.40)	78.70 (11.70)	<0.001
TC (mmol/L), median (IQR)	4.71 (4.15–5.27)	4.37 (3.85–4.92)	<0.001
TG (mmol/L), mean (SD)	1.96 (1.33)	1.74 (1.22)	0.006
HDL-C (mmol/L), mean (SD)	1.20 (0.31)	1.23 (0.30)	0.077
LDL-C (mmol/L), median (IQR)	3.02 (2.60–3.56)	2.73 (2.25–3.23)	<0.001
FBG (mmol/L), mean (SD)	5.86 (1.56)	5.41 (1.07)	<0.001
ALT (U/L), mean (SD)	26.80 (16.80)	25.8 (19.4)	0.324
AST (U/L), mean (SD)	22.20 (9.78)	20.7 (9.25)	0.013
DBIL (µmol/L), mean (SD)	5.06 (1.80)	5.09 (1.96)	0.812
TBIL (µmol/L), mean (SD)	12.50 (5.51)	12.60 (6.16)	0.626
ALP (U/L), mean (SD)	87.90 (23.10)	81.50 (22.50)	<0.001
UA (µmol/L), median (IQR)	324.00 (267.00–385.00)	314.00 (259.00–378.00)	0.208
PLT (10^9^/L), median (IQR)	242.00 (210.00–288.00)	254.00 (216.00–296.00)	0.046
WBC (10^9^/L), mean (SD)	7.88 (2.06)	7.36 (1,96)	<0.001
CRE (µmol/L), mean (SD)	75.00 (11.60)	72.00 (14.20)	<0.001
FLD, *n* (%)			<0.001
Yes	146 (46.9%)	888 (33.6%)	
No	165 (53.1%)	1,757 (66.4%)	
Years of working (years), *n* (%)			<0.001
1–10	23 (7.4%)	874 (33.0%)	
11–20	120 (38.6%)	1,217 (46.0%)	
≥21	168 (54.0%)	554 (20.9%)	
Dust exposure, *n* (%)			0.471
Yes	170 (54.7%)	1,384 (52.3%)	
No	141 (45.3%)	1,261 (47.7%)	
Harmful gas exposure, *n* (%)			0.589
Yes	86 (27.7%)	689 (26.0%)	
No	225 (72.3%)	1,956 (74.0%)	
Alcohol drinking, *n* (%)			<0.001
Yes	132 (42.4%)	792 (29.9%)	
No	179 (57.6%)	1,853 (70.1%)	
Smoke, *n* (%)			<0.001
Yes	185 (59.5%)	978 (37.0%)	
No	126 (40.5%)	1,667 (63.0%)	

HT, height; WT, weight; SBP, systolic blood pressure; DBP, diastolic blood pressure; TC, total cholesterol; TG, triglyceride; HDL-C, high-density lipoprotein cholesterol; LDL-C, low-density lipoprotein cholesterol; FBG, fasting blood glucose; ALT, alanine transaminase; AST, aspartate aminotransferase; DBIL, direct bilirubin; TBIL, total bilirubin; ALP, alkaline phosphatase; UA, uric acid; PLT, blood platelet count; WBC, white blood cell count; CRE, creatinine; FLD, fatty liver disease; Exposure to rock dust and coal dust; Exposure to carbon monoxide and sulfur dioxide.

### 3.2 Screening of features

The correlation statistics chart in the training set demonstrates a robust correlation between the selected features (Supplementary Figure S1). Consequently, three machine learning algorithms were selected to filter the features and subsequently construct the prediction model.

In the XGBoost algorithm, the hyperparameters of the model are selected through cross-validation and random search. The optimal number of iterations of the model is obtained by monitoring the number of iterations of the model using the test data, thereby preventing overfitting (Supplementary Figure S2). The optimal number of iterations and the optimal hyperparameters of the model were then incorporated into the model. The relative importance of the features was determined by training the model on the training set. Features with the highest feature importance were extracted and plotted on a bar graph (Supplementary Figure S3). The five most important features were selected for the next step of model construction, and the resulting prediction model was named the “XGBoost model”.

In the random forest model, the minimum mean squared error and the Gini coefficient are employed as the pivotal hyperparameters in the training set to filter the features. The relative importance of the features is then plotted (only the top ten features are plotted, Supplementary Figure S4). Subsequently, the intersection of the top ten features is taken as the input variable for logistic regression analysis, and the constructed prediction model is designated as the “RF model”.

Finally, LASSO regression was employed to identify the most pertinent features. In the LASSO regression model, the value of *λ* was selected through cross-validation, and the maximum penalty parameter *λ* with the lowest mean square error within one standard deviation was ultimately selected (Supplementary Figure S5). A total of five meaningful variables were obtained under this *λ* for the subsequent model construction, and the constructed predictive model was designated as the “LASSO model”.

### 3.3 Construction and evaluation of the model

**Figure 1:**
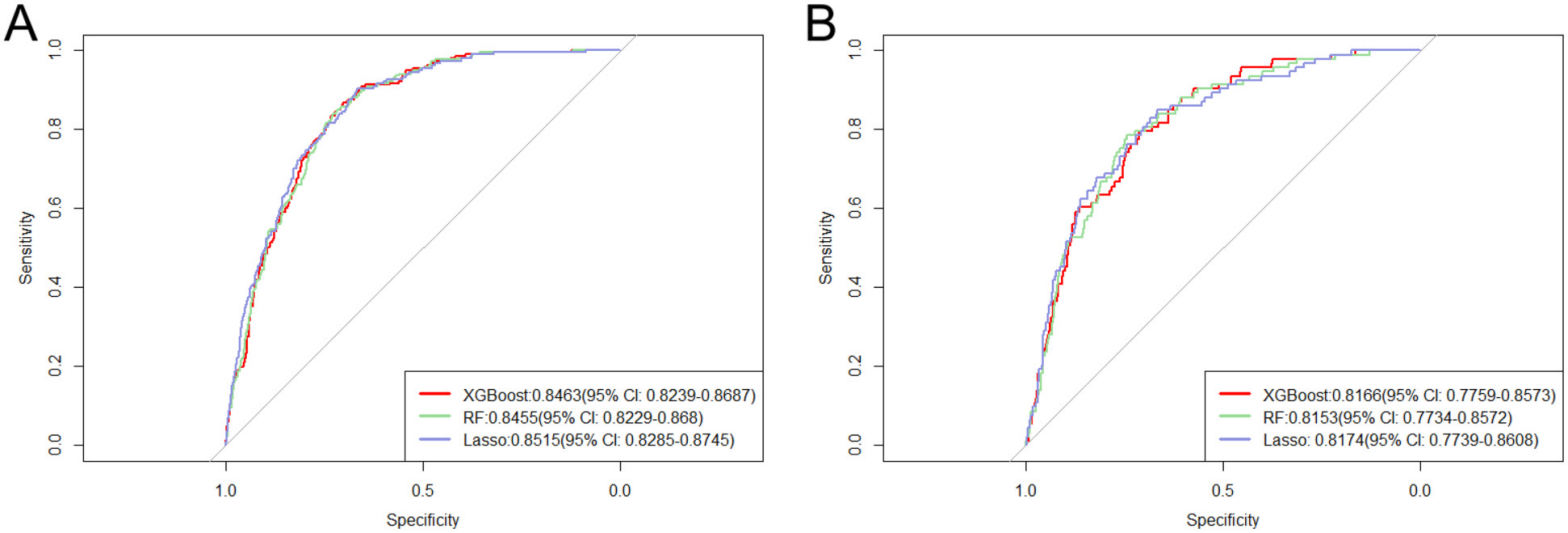
Presents the receiver operating characteristic (ROC) curves for the three models analyzed in the training **(A)** and test **(B)** sets.

Logistic regression models were constructed using features selected by XGBoost, Random Forest and LASSO regression, respectively. The data from the training and test sets were incorporated into the three models to generate their respective ROC curves (Figure 1). The AUCs of the three models in the training set are 0.846, 0.846 and 0.852, respectively. The AUCs of the three models in the test set are 0.817, 0.815 and 0.817, respectively. The AUCs of the “LASSO model” in the training and test sets are higher than those of the other models, although the differences are relatively minor.

The accuracy of the three models in the training cohort and test sets was evaluated by the net reclassification index (NRI) and the integrated discrimination improvement index (IDI), respectively (Table 2). It can be seen that the accuracy of “XGBoost model” is slightly better than the other models. There was a strong correlation between the characteristics of “RF model” and “LASSO model”, including systolic blood pressure (SBP) and diastolic blood pressure (DBP), total cholesterol (TC) and low-density lipoprotein cholesterol (LDL-C) (Supplementary Figure S1). The inclusion of variables with strong correlations in a predictive model can lead to an increase in the variance of parameter estimates, which in turn reduces predictive accuracy. In severe cases, anomalies may occur in which the predictive model does not reflect the relationship between the input and output variables, but rather their joint influence on the output variables. Therefore, the “XGBoost model” is chosen as the final model in this study, and the formula of the model is shown below:

$$P=\frac{e^{-13.5554+0.1544\times \mathrm{Age}+0.0349\times \mathrm{DBP}+0.3557\times \mathrm{HDL}-C+0.0754\times \mathrm{WBC}+0.0092\times \mathrm{BMI}}}{1+e^{-13.5554+0.1544\times \mathrm{Age}+0.0349\times \mathrm{DBP}+0.3557\times \mathrm{HDL}-C+0.0754\times \mathrm{WBC}+0.0092\times \mathrm{BMI}}}$$

**Table 2:** Presents an assessment of the accuracy of the model, as measured by the Net Reclassification Index (NRI) and the Integrated Discriminant Improvement Index (IDI).

Comparison model	NRI	*P* value	IDI	*P* value
Training set				
XGB model vs. RF model	−0.001	0.939	0.027	0.211
XGB model vs. LASSO model	0.004	0.803	0.001	0.948
RF model vs. LASSO model	0.006	0.752	−0.026	0.461
Test set				
XGB model vs. RF model	0	1	0.015	0.599
XGB model vs. LASSO model	0.021	0.461	0	1
RF model vs. LASSO model	0.021	0.461	0.041	0.595

**Figure 2:**
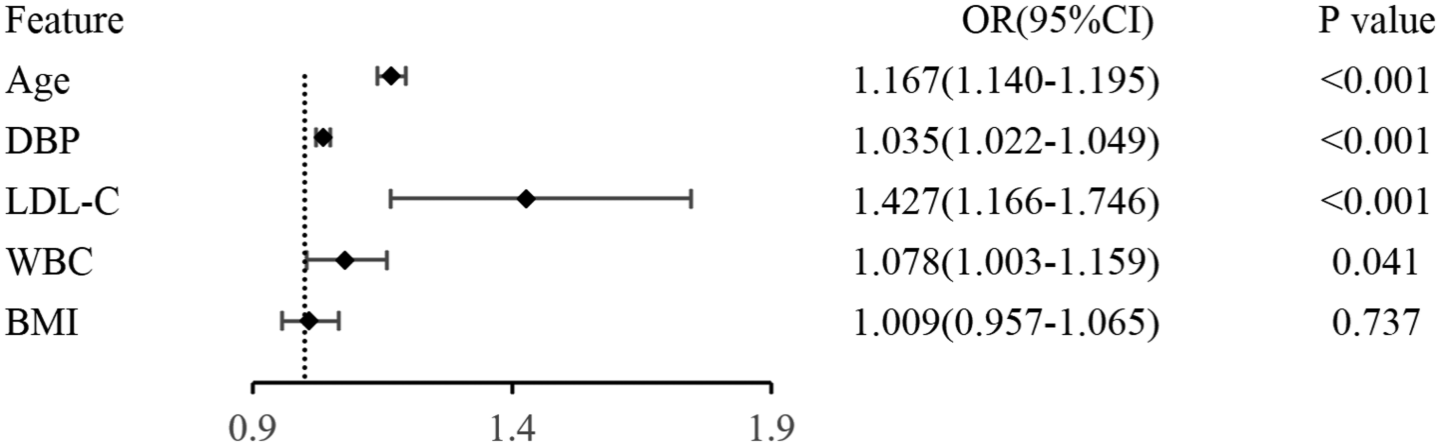
Presents the construction of a forest plot based on the features of the “XGBoost model”.

A forest plot was constructed based on the characteristics of the “XGBoost model” (Figure 2). It was found that age, diastolic blood pressure (DBP), low-density lipoprotein cholesterol (LDL-C), and white blood cell count (WBC) were the risk factors for carotid artery plaques among coal miners, with odds ratios (ORs) greater than 1 and statistically significant differences.

### 3.4 Visualization of the prediction model and evaluation of its effectiveness

**Figure 3:**
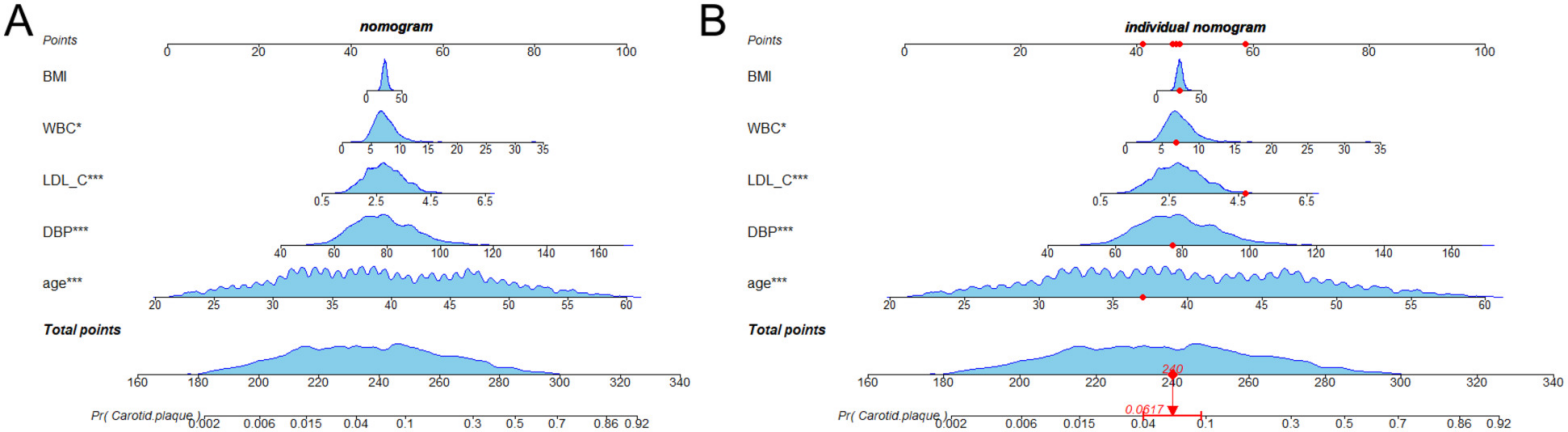
Nomogram for predicting carotid artery plaque in miners. The blue areas in the plots indicate the distribution of variables in each feature. **(A)** Nomogram constructed based on the data in the training set; **(B)** Case nomogram plotted using the first sample in the training set as an example.

A nomogram was constructed using the features from the XGBoost model to assess the risk of carotid plaque in miners (Figure 3A). Using the first sample in the training set as an example, a nomogram was created to locate the risk score for age (Figure 3B). A straight line was drawn vertically on the “scores” axis to determine how many scores correlate with the risk for age. This process is repeated for each trait, with the sum placed on the “total points” axis. Finally, a straight line is drawn vertically down to give the risk of carotid plaque in miners.

**Figure 4:**
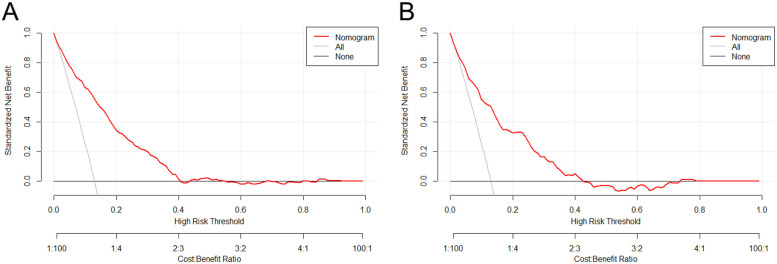
Presents the decision curve analysis of the nomogram in the training set **(A)** and the test set **(B)** the horizontal coordinates represent the probability thresholds. The line labeled “None” indicates the net clinical gain curve if all patients are not intervened. The line labeled “All” is the net clinical gain curve if all patients are intervened. The red line represents the net benefit curve for “treating” patients within each prediction threshold in the training (or test) set. The bottom horizontal line represents the loss: benefit ratio, which represents the proportion of loss and benefit at different probability thresholds.

**Figure 5:**
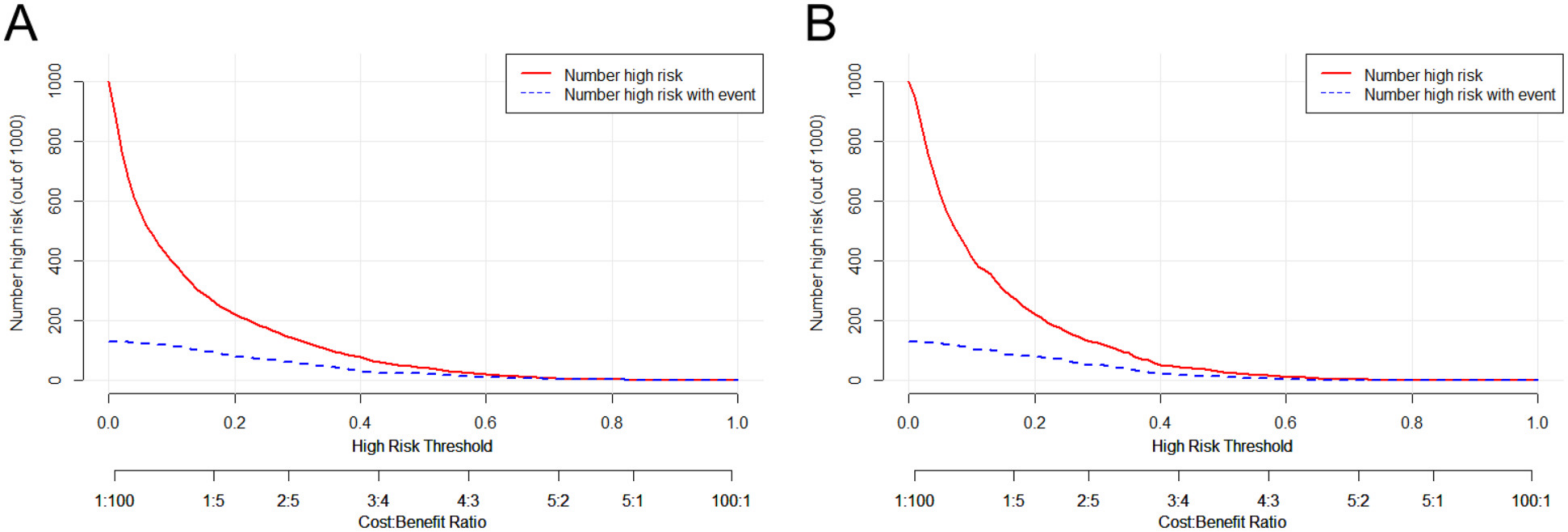
Presents the clinical impact curve analysis of the nomogram in the training set **(A)** and test set **(B)** the horizontal coordinates represent the probability thresholds, while the vertical coordinates indicate the number of individuals. The red line illustrates the number of individuals predicted by the model to be at high risk at different probability thresholds, while the blue line depicts the number of individuals predicted to be at high risk who actually experienced an outcome event at different probability thresholds. The bottom horizontal line represents the loss: gain ratio, which indicates the proportion of losses and gains at different probability thresholds.

In parallel, the decision curve analysis (DCA) and clinical impact curve (CIC) of the nomogram were plotted on the training and test sets. The prevalence of carotid plaque in miners was found to be approximately 13.06% in the previous study, thus serving as the baseline prevalence of carotid plaque in miners to plot the DCA and CIC. The analysis of the decision curves revealed that the threshold range of the nomogram in the training set was 0.02–0.40, with the highest net gain of 0.88 (Figure 4A). In the test set, the threshold range was 0.03–0.42, with the highest net gain of 0.82 (Figure 4B). The results of the DCA were used to plot clinical impact curves, which were employed to assess the clinical utility of the nomogram. The results of the clinical impact curves demonstrated that the predicted probabilities were in good agreement with the actual probabilities (Figure 5A), and similar results were obtained in the test set (Figure 5B).

## 4 Discussion

In this study, a predictive model was developed to predict the risk of carotid plaque in coal miners. Three machine learning algorithms were employed to filter the features, and based on the filtered features, a predictive model was constructed using logistic regression. Following the comparison of the three models, the XGBoost model was identified as the most effective, with an AUC, sensitivity and specificity of 0.846, 0.867 and 0.702, respectively. This may be attributed to the XGBoost approach, which combines the prediction results of multiple weak learners (decision trees) to construct a more effective model, ultimately improving overall performance. Furthermore, XGBoost employs early “stops” to prevent overfitting, thereby enhancing its generalizability. Finally, a nomogram has been constructed based on the features of the “XGB model”, facilitating the prediction probability of individual samples.

In comparison to other studies, our research team developed a carotid plaque risk prediction model that applies to coal miners. This model included a greater number of characteristics (18, 19), such as years of work experience, dust exposure, exposure to hazardous gases, and lifestyle habits. All of these characteristics are relevant to miners, and although they were not included in the final prediction model, the differences between healthy individuals and patients with carotid plaques were statistically significant. This suggests that these characteristics may provide food for thought for future studies. Xie et al. identified a significant association between air pollution and carotid plaque using a COX proportional risk model (12), whereas other studies did not include this feature. Coal miners are often exposed to hazardous gases such as dust, carbon monoxide and hydrogen sulphide. Sugiura et al. employed multivariate logistic regression to ascertain an association between unhealthy lifestyle habits, such as habitual smoking, and atherosclerosis (14); other studies did not adjust for lifestyle characteristics.

The predicted probabilities of the three prediction models exhibited minimal discrepancy (the difference between the predictions was not statistically significant). We identified the features that appeared twice in the three models as the most crucial features, which were age, systolic blood pressure, diastolic blood pressure, total cholesterol and low-density lipoprotein cholesterol. Among these, age, as the most crucial feature, demonstrated a robust correlation in all three models, which is consistent with previous findings (20). Hypertension has been demonstrated to elevate the risk of carotid plaque formation, a finding consistent with our own observations (21, 22). However, a study conducted in a middle-aged and elderly population indicated that systolic and diastolic blood pressure exert distinct effects on carotid plaque, with one being a risk factor for plaque development and the other a protective factor (23). This is contrary to the previously held view that systolic blood pressure is a risk factor for carotid plaque formation. This discrepancy may be attributed to the fact that systolic blood pressure tends to increase with age (24), while diastolic blood pressure tends to decrease (25), which is considered a protective factor. In contrast, the present study's main population consisted of miners undergoing a physical examination, who were considerably younger and in better physical condition. The relationship between diastolic blood pressure and carotid plaque remains unknown. Our findings may provide insight into this relationship. Additionally, our study found that elevated blood lipids (e.g., total cholesterol and low-density lipoprotein cholesterol) were associated with the prevalence of carotid plaque, consistent with previous studies (26, 27). Furthermore, we included BMI and white blood cell count (WBC) as predictors in the XGBoost model. Elevated BMI is an important risk factor for carotid plaque formation, as it can increase peripheral vascular resistance, which in turn promotes plaque formation (28, 29). One study has shown that the arteries of obese individuals begin to harden during adolescence (30), which emphasizes the importance of maintaining a normal BMI. A number of epidemiological studies have demonstrated a correlation between inflammatory biomarkers (e.g., white blood cell count) and the formation of carotid plaques (31, 32). This is consistent with our findings. Furthermore, inflammation has been identified as a risk factor for carotid plaque even in individuals with a normal weight and a healthy metabolic profile (33).

In this study, we constructed a nomogram for the prediction of carotid plaque risk in miners based on the features identified by the “XGBoost model”. We then introduced the decision curve analysis (DCA) and the clinical impact curve (CIC) to evaluate the performance of the nomogram. The CIC is the weighted average of the absolute mean difference between the observed probability and the predicted probability. It can be used to quantitatively evaluate the results of binary classification and thus provide a more comprehensive evaluation of the effect of the nomogram (34).

This study is subject to several limitations. Firstly, the data used to train the model in this study was derived from the physical examination of miners, which is cross-sectional in nature and therefore unable to demonstrate a causal relationship between traits and diseases. Secondly, the features encompassed in this study remain insufficiently comprehensive. For instance, prior studies have demonstrated that night shift work is correlated with an elevated risk of carotid plaque (35), and night shift work is highly prevalent among miners. Night shift work is likely to have a direct bearing on miners' health; the specific occupations of miners, such as coal miners, electricians, and ventilation workers, are associated with exposure to different levels of harmful gases and dust, which may seriously affect their health; therefore, failure to consider these factors may lead to biased research results. Thirdly, the research population chosen in this study pertains specifically to coal miners in a particular area, which might have an impact on the generalization of the results. The fact that the working conditions and living environments of coal miners in this area could differ from those in other regions or workers with diverse occupational backgrounds constrains the universality and generalizability of the research findings. Fourth, the dataset used in this study exhibits a significant class imbalance, with a much lower number of positive cases compared to negative cases. While this imbalance reflected real-world clinical scenarios and ensured the model's robustness, it may also pose challenges for model training and performance evaluation. Future studies could contemplate including night shift work as an independent variable and conducting research among coal miners in various regions, with distinct working conditions and living habits, to enhance the diversity of the sample and the representativeness of the research results. Additionally, future work may explore data balancing methods to further optimize model performance.

## 5 Conclusions

In this study, we employed three machine learning methods to screen for features and constructed a predictive model for carotid plaque risk in coal miners using logistic regression. The XGBoost algorithm demonstrated the most effective performance in the screening of features, with an AUC, sensitivity, and specificity of 0.846, 0.867, and 0.702, respectively. This method contributes to the personalized risk assessment of carotid plaque in coal miners and has the potential to enhance the cost-effectiveness of carotid ultrasound testing.

## Data Availability

The original contributions presented in the study are included in the article/Supplementary Material, further inquiries can be directed to the corresponding authors.
