# Supplementary material for: Development and validation of a carotid plaque risk prediction model for coal miners
Source: Front Cardiovasc Med. 2025 May 9;12:1490961. doi: 10.3389/fcvm.2025.1490961 (PMC12098412; doi:10.3389/fcvm.2025.1490961)
Supplement: Supplementary file 1 [file Datasheet1.pdf]

Supplementary Material

1 Supplementary Figures and Tables

1.1 Supplementary Tables

Supplementary Table 1. Presents the characteristics of the study subjects in the training set

Characteristic	Training cohort		
	Carotid plaque		P value
	Yes(n=218)	No（n=1851）	
Sex, n(%)			<0.001
Female	20 (9.2%)	534 (28.8%)	
Male	198 (90.8%)	1317 (71.2%)	
Age(years), mean (SD)	47.70(6.39)	38.50(7.81)	<0.001
HT (cm), median (IQR)	169.90(164.90-174.03)	170.00(164.30-175.00)	0.724
WT (kg), median (IQR)	76.05 (68.38-82.93)	73.60 (65.00-82.10)	0.006
BMI (kg/m^2), median (IQR)	26.38 (24.64-28.12)	25.46 (23.24-27.77)	0.001
SBP (mm/Hg), mean (SD)	143.00 (19.10)	131.00 (16.30)	<0.001
DBP (mm/Hg), mean (SD)	86.40 (13.70)	78.30 (11.40)	<0.001
TC (mmol/L), median (IQR)	4.71 (4.16-5.28)	4.36 (3.84-4.92)	<0.001
TG (mmol/L), mean (SD)	1.98(1.45)	1.74(1.26)	0.020
HDL-C (mmol/L), mean (SD)	1.21(0.33)	1.24(0.31)	0.265
LDL-C (mmol/L), median (IQR)	3.02(2.61-3.54)	2.73(2.25-3.23)	<0.001
FBG (mmol/L), mean (SD)	5.91(1.66)	5.41(1.10)	<0.001
ALT (U/L), mean (SD)	26.50(15.30)	25.90(19.50)	0.595

AST (U/L), mean (SD)	21.90(9.56)	20.70(9.00)	0.079
DBIL (μmol/L), mean (SD)	5.10(1.76)	5.06(1.98)	0.771
TBIL (μmol/L), mean (SD)	12.50(5.21)	12.60(6.22)	0.810
ALP (U/L), mean (SD)	87.70(22.50)	81.40(22.30)	<0.001
UA (μmol/L), median (IQR)	323.00 (268.00-382.50)	304.00 (255.00-382.25)	0.246
PLT (10^9/L), median (IQR)	242.00 (210.75-285.00)	255.00 (220.00-298.25)	0.061
WBC (10^9/L), mean (SD)	7.82(2.02)	7.32(1.94)	0.001
CRE (μmol/L), mean (SD)	74.70(11.00)	71.60(14.80)	<0.001
FLD, n (%)			0.001
Yes	615 (33.2%)	98 (45.0%)	
No	120 (55.0%)	1236 (66.8%)	
Years of Working(years), n (%)			<0.001
1-10	16 (7.3%)	629 (34.0%)	
11-20	92 (42.2%)	825 (44.6%)	
≥21	110 (50.5%)	397 (21.4%)	
Dust Exposure, n (%)			0.027
Yes	132 (60.6%)	970 (52.4%)	
No	86 (39.4%)	881 (47.6%)	
Harmful Gas Exposure, n (%)			0.278
Yes	67 (30.7%)	500 (27.0%)	
No	151 (69.3%)	1351 (73.0%)	
Alcohol Drinking, n (%)			<0.001
Yes	94 (43.1%)	554 (29.9%)	

No	124 (56.9%)	1297 (70.1%)	
Smoke, n (%)			<0.001
Yes	131 (60.1%)	670 (36.2%)	
No	87 (39.9%)	1181 (63.8%)	

HT , height; WT, weight; SBP, systolic blood pressure; DBP, diastolic blood pressure; TC, total cholesterol; TG, triglyceride; HDL_C, high-density lipoprotein cholesterol; LDL_C, low-density lipoprotein cholesterol; FBG, fasting blood glucose; ALT, alanine transaminase; AST, aspartate aminotransferase; DBIL, direct bilirubin; TBIL, total bilirubin; ALP, alkaline phosphatase; UA, uric acid; PLT, blood platelet count; WBC, white blood cell count ;CRE, creatinine; FLD, fatty liver disease; Exposure to rock dust and coal dust; Exposure to carbon monoxide and sulfur dioxide.

Supplementary Table 2. Presents the characteristics of the study subjects in the validation set

Characteristic	Validation cohort		P value
	Carotid plaque		
	Yes(n=93)	No（n=794）	
Sex, n (%)			0.023
Female	14 (15.1%)	210 (26.4%)	
Male	79 (84.9%)	584 (73.6%)	
Age(years), mean (SD)	47.10(6.86)	38.90(7.55)	<0.001
HT (cm), median (IQR)	169.50(164.85-174.10)	170.50(164.70-175.50)	0.303
WT (kg), median (IQR)	75.10 (67.90-84.05)	74.10 (64.30-83.10)	0.311
BMI (kg/m2), median (IQR)	25.91 (24.02-28.88)	25.64 (23.12-27.92)	0.070
SBP (mm/Hg), mean (SD)	142.00 (18.80)	132.00 (17.40)	<0.001
DBP (mm/Hg), mean (SD)	85.30 (12.70)	79.60 (12.20)	<0.001
TC (mmol/L), median (IQR)	4.75 (4.13-5.26)	4.41 (3.85-4.92)	0.005

TG (mmol/L), mean (SD)	1.91(0.98)	1.75(1.11)	0.141
HDL_C (mmol/L), mean (SD)	1.18(0.26)	1.23 (0.30)	0.094
LDL_C (mmol/L), median (IQR)	3.00(2.59-3.63)	2.72(2.27-3.24)	0.004
FBG (mmol/L), mean (SD)	5.73(1.30)	5.42(1.00)	0.025
ALT (U/L), mean (SD)	27.60(20.10)	25.60(19.30)	0.369
AST (U/L), mean (SD)	22.70(10.30)	20.60(9.83)	0.072
DBIL (μmol/L), mean (SD)	4.97(1.89)	5.15(1.90)	0.404
TBIL (μmol/L), mean (SD)	12.50(6.20)	12.80(6.02)	0.626
ALP (U/L), mean (SD)	88.40(24.50)	81.70(23.00)	0.013
UA (μmol/L), median (IQR)	326.00 (259.50-388.00)	315.50 (264.00-383.25)	0.590
PLT (109/L), median (IQR)	241.00 (206.00-290.50)	255.50 (216.00-293.25)	0.443
WBC (109/L), mean (SD)	8.01(2.15)	7.46 (2.02)	0.020
CRE (μmol/L), mean (SD)	75.90(12.80)	72.80(12.60)	0.033
FLD, n (%)			0.002
Yes	48 (51.6%)	273 (34.4%)	
No	45 (48.4%)	521 (65.6%)	
Years of Working(years), n (%)			<0.001
1-10	7 (7.53%)	245 (30.9%)	
11-20	28 (30.1%)	392 (49.4%)	
≥21	58 (62.4%)	157 (19.8%)	
Dust Exposure, n (%)			0.051
Yes	38 (40.9%)	414 (52.1%)	
No	55 (59.1%)	380 (47.9%)	

Harmful Gas Exposure, n (%)			0.550
Yes	19 (20.4%)	189 (23.8%)	
No	74 (79.6%)	605 (76.2%)	
Alcohol Drinking, n (%)			0.043
Yes	38 (40.9%)	238 (30.0%)	
No	55 (59.1%)	556 (70.0%)	
Smoke, n (%)			0.001
Yes	54 (58.1%)	308 (38.8%)	
No	39 (41.9%)	486 (61.2%)	

HT , height; WT, weight; SBP, systolic blood pressure; DBP, diastolic blood pressure; TC, total cholesterol; TG, triglyceride; HDL_C, high-density lipoprotein cholesterol; LDL_C, low-density lipoprotein cholesterol; FBG, fasting blood glucose; ALT, alanine transaminase; AST, aspartate aminotransferase; DBIL, direct bilirubin; TBIL, total bilirubin; ALP, alkaline phosphatase; UA, uric acid; PLT, blood platelet count; WBC, white blood cell count ;CRE, creatinine; FLD, fatty liver disease; Exposure to rock dust and coal dust; Exposure to carbon monoxide and sulfur dioxide.

1.2 Supplementary Figures

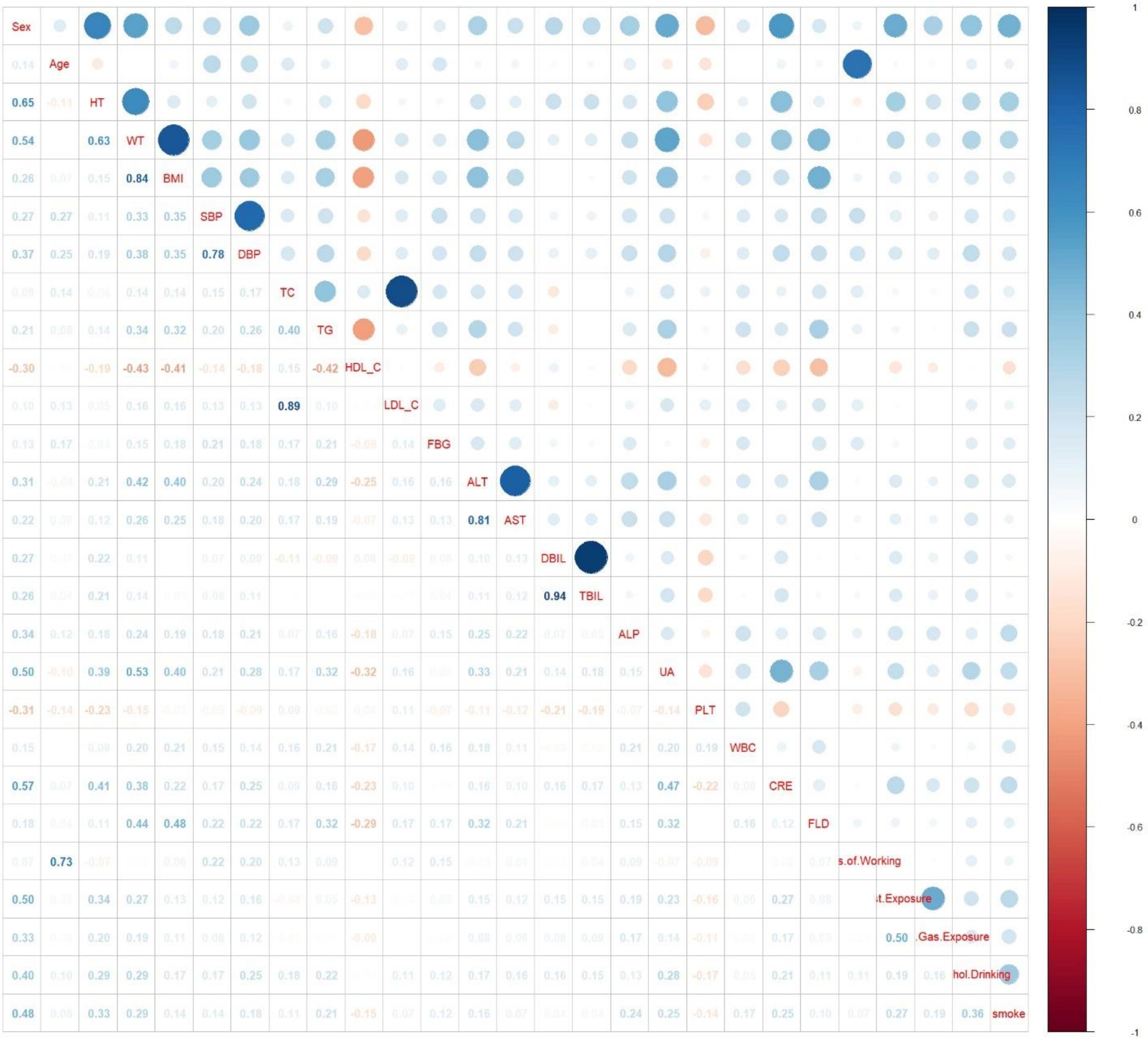

Supplementary Figure 1. Correlation statistics of all features in the training set

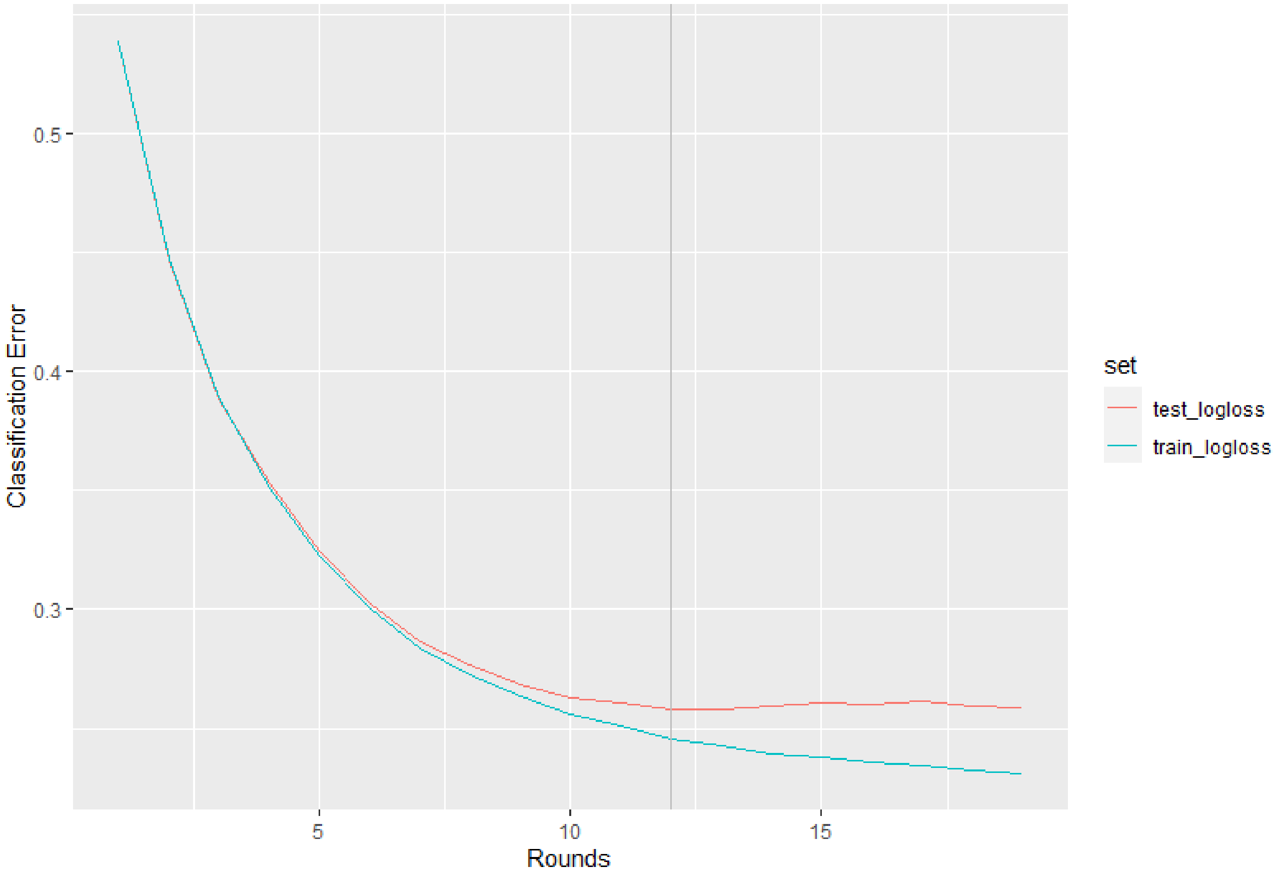

Supplementary Figure 2. Optimal iteration rounds for XGBoost. The data in the training set is reclassified into training data and test data. The training data is used to fit the model. When the classification error rate in the training data continues to decrease while the classification error rate in the test data increases, it can be concluded that the model is at risk of overfitting. In such a case, it is advisable to stop the training process at an early stage.

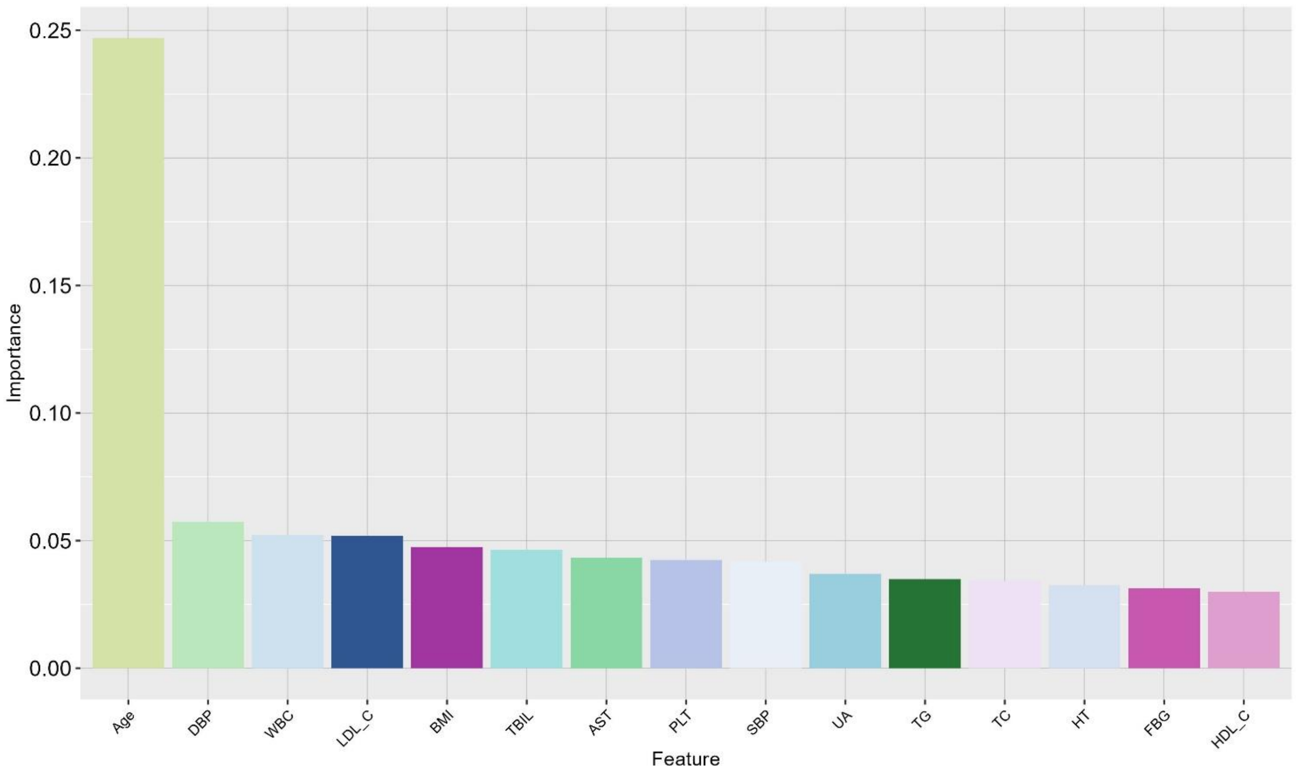

Supplementary Figure 3. Presents the relative importance of features in the training set for the XGBoost model

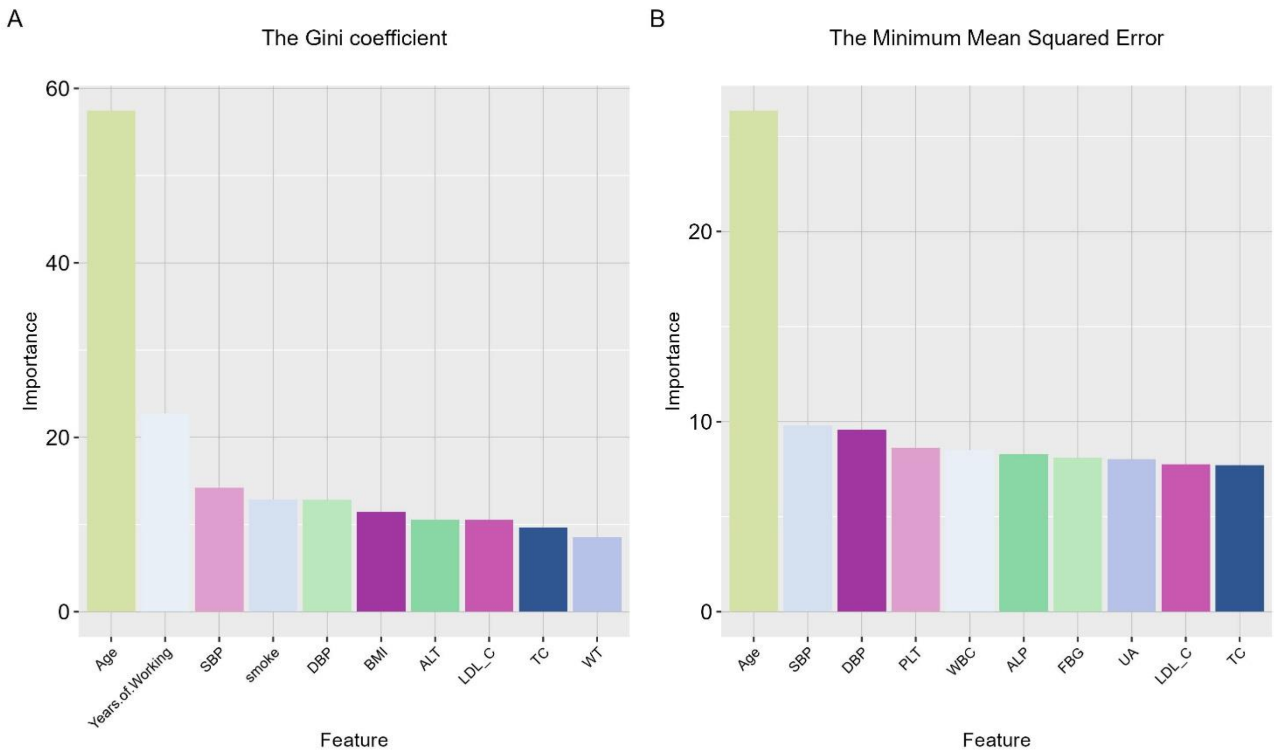

Supplementary Figure 4. Presents the relative importance of features in the Random Forest model on the training set. (A) Features were screened using the Gini coefficient as the main parameter, with the horizontal coordinate measuring the magnitude of the gain, i.e., the increase in the purity of the node, from adding the variable to the node. The higher the Mean Decrease Gini, the more important the variable is, and vice versa, the less important it is. (B)The horizontal coordinate indicates the amount of increase in the average Mean Decrease Accuracy compared to the full model when a feature is removed. Features are screened using the Minimum Mean Decrease Accuracy as the main parameter. The higher the Mean Decrease Accuracy, the more important the variable is, and vice versa for unimportance.

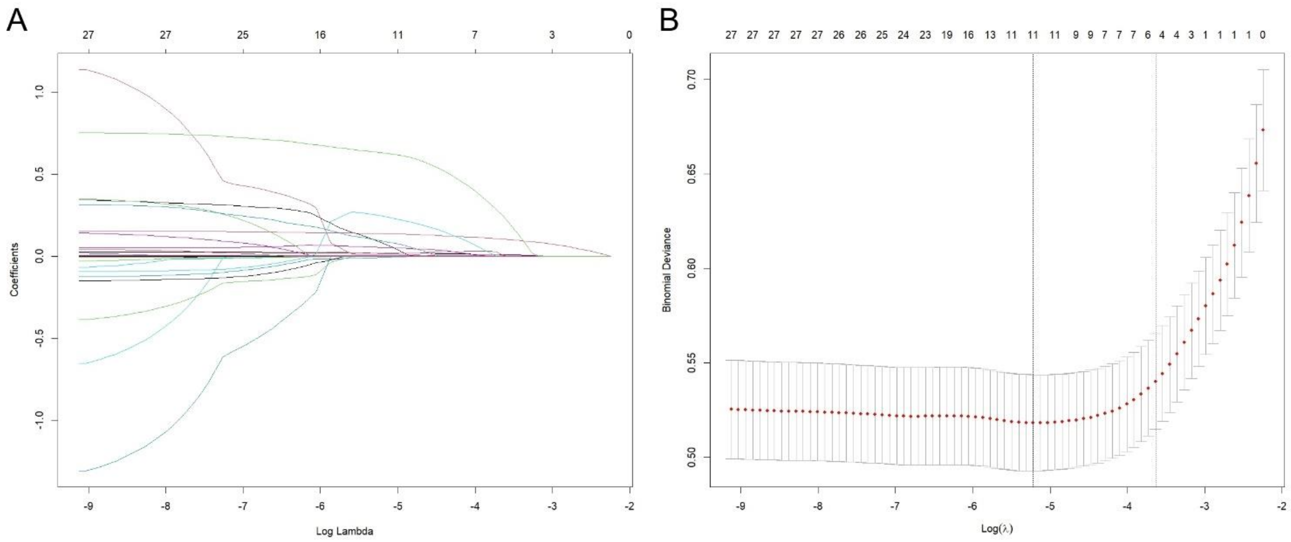

Supplementary Figure 5. the application of LASSO regression for the purpose of screening the features of the predictive model. (A)The figure depicts the coefficient distribution of the LASSO regression, which allows for the observation of the trajectory of carotid plaque-related features as a function of the LASSO algorithm parameter, λ. (B)The figure illustrates the ten-fold cross-validation process in the training set, which was employed to determine the optimal penalty coefficient λ.
